# Effect of Ultrasonic-Assisted Casting on the Hydrogen and Lithium Content of Al-Li Alloy

**DOI:** 10.3390/ma15031081

**Published:** 2022-01-30

**Authors:** Yuqi Hu, Ripeng Jiang, Xiaoqian Li, Renjun Hu

**Affiliations:** 1 Research Institute of Light Alloy, The College of Mechanical and Electrical Engineering CMEE, Central South University, No. 932 Lushan Road (S), Main Campus, Changsha 410083, China; hyqalbert@csu.edu.cn (Y.H.); meel@csu.edu.cn (X.L.); renjunhu1026@csu.edu.cn (R.H.); 2 State Key Laboratory of High-Performance Complex Manufacturing, Changsha 410083, China

**Keywords:** ultrasonic degassing, hydrogen and lithium content, tensile properties

## Abstract

Dehydrogenation of the 2195 Al–Li alloy was accomplished using argon degassing, ultrasonic degassing, and vacuum degassing. The concentration of hydrogen, its microstructure, and its mechanical characteristics were all investigated. The hydrogen content in the 2195 Al–Li alloy is high. The degassing process significantly improved the mechanical properties of the cast alloy, owing the removal of hydrogen. Among the three degassing techniques, ultrasonic argon treatment was an efficient dehydrogenation approach and an effective procedure for enhancing the microstructure while minimizing lithium loss in the Al–Li alloy. On the one hand, ultrasonic waves can dissolve purged argon bubbles, allowing them to degas more efficiently. On the other hand, ultrasonic waves may cause a large number of cavitation bubbles to form in the melt, which should be the cause of the microstructure refinement. The dynamics of rising argon bubbles and ultrasonic effects are involved in ultrasonic argon treatments such as cavitation and flow.

## 1. Introduction

Hydrogen content is one of the indicators of an aluminum alloy’s casting quality, and its presence reduces the mechanical properties of cast products. During the solidification process, hydrogen may be released from the solution, or the difficulty of feeding liquid metal through the interdendritic area may cause porosity in the casting [1]. The only gas that can dissolve in molten aluminum is hydrogen [2]. As a result, in foundries, the control of dissolved hydrogen in the melt is highly challenging. Degassing is the most efficient method for lowering hydrogen porosity. Some of the methods used include nitrogen, argon, or a combination of both, as well as chlorine and hexachlorophene (C2Cl6) tablets [3]. Vacuum [4] and ultrasonic [5] degassing can be employed separately or in combination.

Ultrasonic degassing creates fluctuating pressure fields in molten aluminum by using high-intensity vibrations. As a result, the liquid’s alternating pressure fields form a large number of small holes. Some of these cavities form quickly under the influence of alternating pressure and unidirectional diffusion of the dissolved hydrogen into the accessible cavities, resulting in gas bubbles that agglomerate and float to the melt’s surface, and the hydrogen finds a path to escape [6].

The process of ultrasonic degassing has been linked to cavitation, which is dependent on ultrasonic strength, according to certain studies [7]. The ultrasonic degassing technology is currently gaining popularity due to its environmental friendliness and resource efficiency. Some scientists have looked into it. The mechanism of ultrasonic degassing was discovered to be attributed to cavitation, which is dependent on the power of ultrasonics. The results suggest that raising the temperature or the treatment time might improve the degassing impact [8]. The degassing effect in molten or semisolid slurry aluminum alloys was thought to be a combination of cavitation and ultrasound acoustic streaming, which was related to ultrasonic power, according to Wu et al. [9]. Many parameters influence the efficiency of ultrasonic degassing, including power, ultrasonic duration, treatment temperature, and resonance or non-resonance [10]. As a result, optimizing ultrasonic degassing settings for industrial aluminum alloys is critical.

Due to the special requirements of the Al–Li alloy casting process for melting and casting conditions, the combination of argon gas and ultrasonic degassing is an effective approach [11]. For significant volumes of melt, ultrasonic-aided degassing appears to be a viable option. Ultrasonic vibration causes cavitation bubbles, which break big bubbles into smaller bubbles that are equally scattered in the melt during ultrasonic degassing. Thus, in this study, ultrasonic-assisted degassing of Al–Li alloy ingots was investigated and compared with typical argon and vacuum degassing procedures.

## 2. Materials and Methods

**Figure 1:**
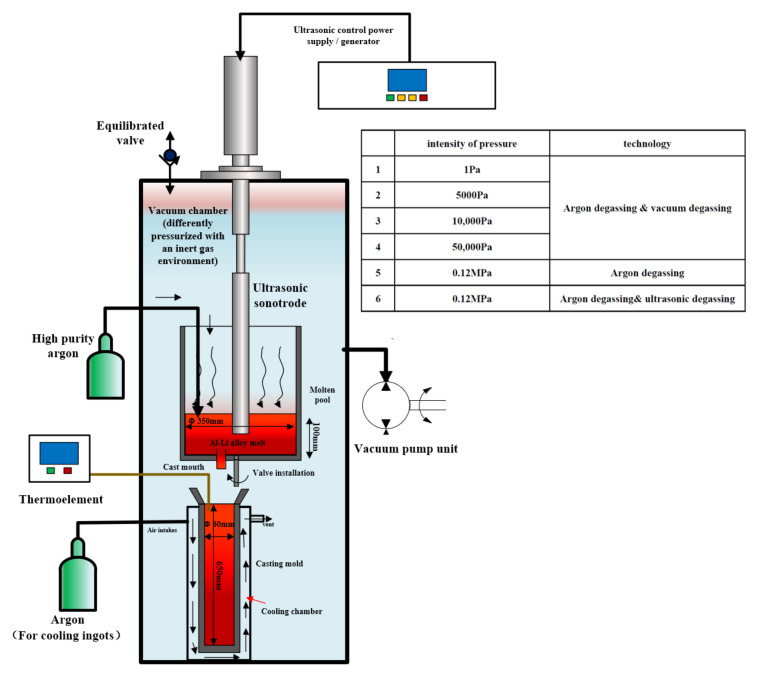
The schematic for the equipment.

The ultrasonic resonant transducer used a frequency of 20 kHz and an input power of 12 kW. An experiment was carried out in a crucible of 2195 Al–Li alloy weighing 7 kg. As seen in Figure 1, the heating furnace and the casting mold were placed in a vacuum chamber, the molten pool in the heating furnace had a diameter of Φ350 mm and a height of 100 mm, the size of the casting mold was Φ60–650 mm, and a cooling chamber was arranged in the casting mold for cooling the ingot. The device had a connected ultrasonic transducer. When the transducer was turned on, the ultrasonic sonotrode immersed in the melt sent ultrasonic waves into the melt, which propagated in the melt. The argon injection rate was set at 0.17 m^3^/h, and the ventilation time was 10 min in this experiment, which was controlled using a flow meter. The melt was kept at a constant temperature, and bubbles were allowed to rise to the surface. At this time, the vacuum pump was turned on to adjust the vacuum in the cavity, and different vacuum conditions were introduced (10 Pa, 5000 Pa, 0.01 MPa, 0.05 MPa, and 0.1 MPa (atmospheric pressure)). At the same time, under the condition of 0.1 MPa, the ultrasonic equipment was turned on, with an ultrasonic vibration time of 15 min. The control ingot underwent no degassing treatment at 0.1 MPa. Casting was performed through the cast mouth and valve without changing the pressure in the vacuum chamber. Finally, the casting mold was cooled under the same flow rate of argon cooling until it reached below 100 °C. Seven ingots with a size of Φ60–650 mm and a relatively large length and diameter were obtained. This study used the 2195 Al–Li alloy; the element composition is described in Table 1.

Standard metal procedures were used to grind and polish all of the samples. A scanning electron microscope was used to examine the microstructure (SEM: TESCAN, MIRA3 LMH/LMU, Carl Zeiss Shanghai, China) Co., Ltd., along with a spectrometer for energy-dispersive spectroscopy to analyze the composition of the sample. To meet the requirements of metal phase inspection, all of the samples were treated with Barker’s reagent. The metallic phase observation was performed under polarized light, and the ZEISS optical microscope was equipped with an automatic Zeiss AxioVision image analyzer (Carl Zeiss, Shanghai, China). The particle size was analyzed according to the linear intercept method (ASTM E112-10).

**Figure 2:**
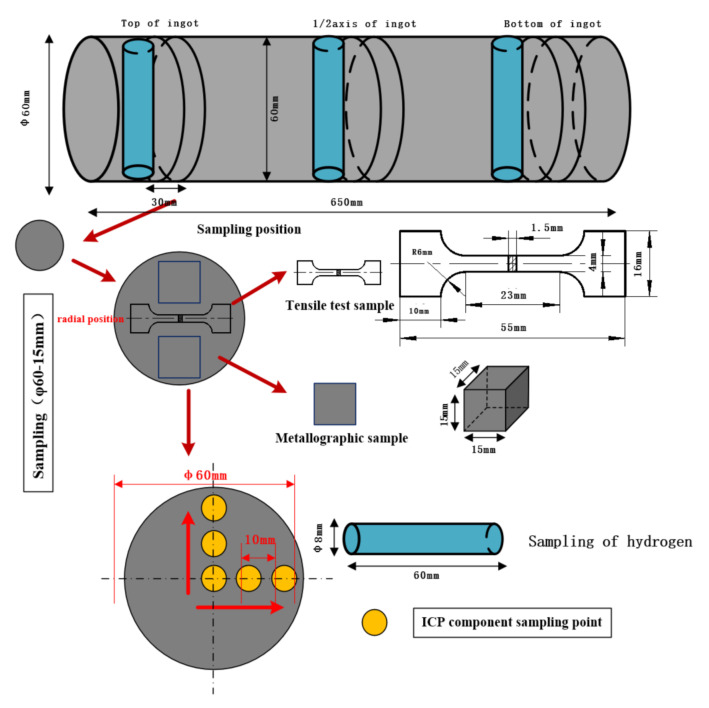
The schematic for sample preparation.

The solid hydrogen content of the three alloy ingots was tested. The test equipment used was a high-precision RHEN602 hydrogen analyzer. The sampling method is shown in Figure 2. Before testing the hydrogen content, a small lathe was used to test an ingot of Φ8–60 mm. The hydrogen measurement samples were evenly divided into three sections to remove the oxide scale on the surface of the samples, and then quickly soaked in acetone solution to improve the accuracy of the test and reduce the error value during the experiment. Separate detection was applied to determine the average value of hydrogen content at specific positions of the ingot.

## 3. Results

**Figure 3:**
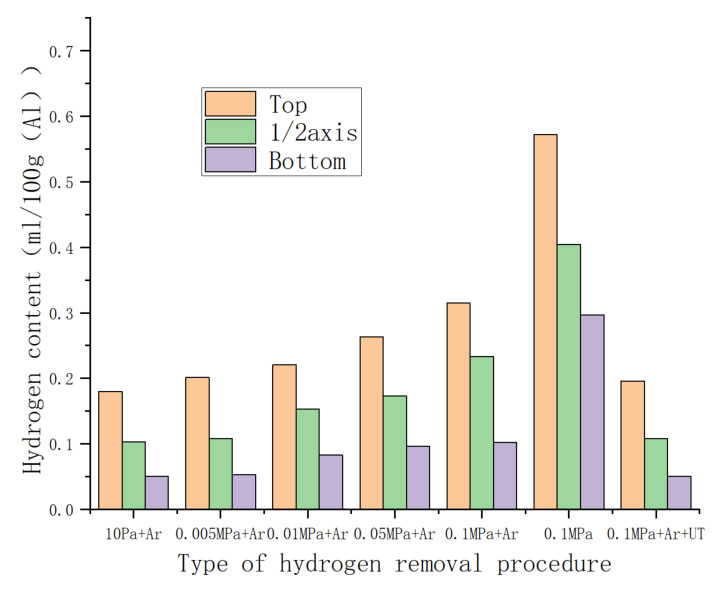
The content of Li at the axial sampling position of the Φ60 mm 2195 Al–Li alloy ingot.

The hydrogen concentration in the melt was greatly reduced under low pressure, and its hydrogen content value was 0.111 mL/100 g (Al) under 10 Pa, according to the statistics of hydrogen content in each location of the conventional 2195 Al–Li alloy ingot under different casting circumstances in Figure 3. The gas on the melt interface was eliminated, and the gas in the melt was simpler to extract from the melt during the refining process under low-pressure circumstances. The moisture in the high-purity argon reacted with the lithium element in the melt as the pressure rises, increasing the hydrogen concentration of the melt. Because of cavitation, ultrasonic treatment is well recognized for its ability to both degas and purify the melt [13,14]. When both ultrasound and argon were used at the same time, the results were impressive. Vacuum degassing was less effective than ultrasonic degassing but was more efficient than single argon degassing.

**Figure 4:**
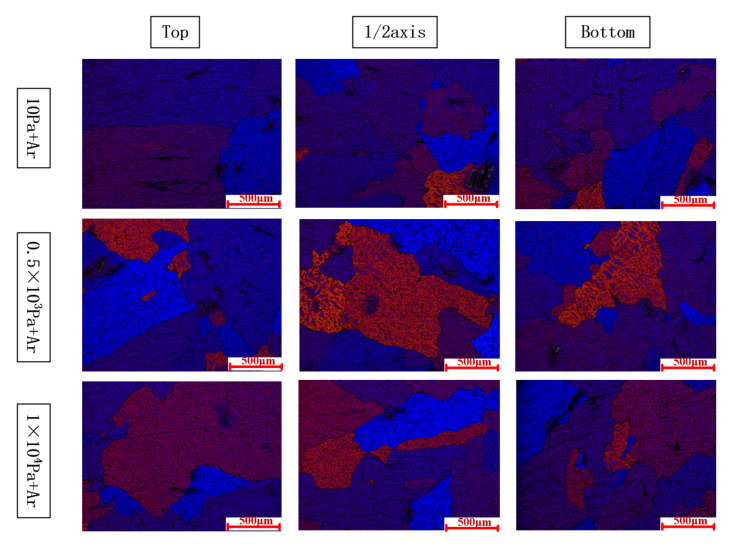

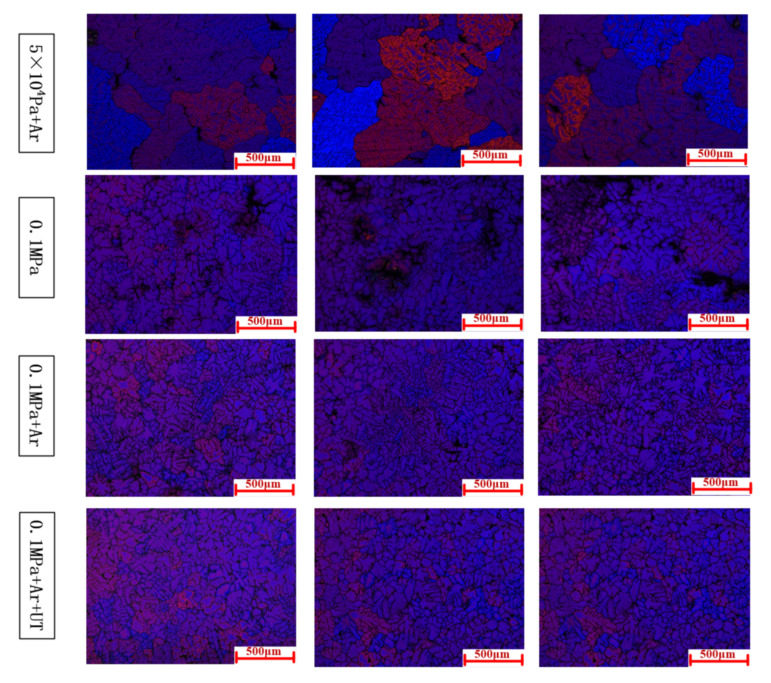
Optical micrographs of the AA2195 under different hydrogen removal procedures.

Figure 4 shows the results of a metallographic microscope at different parts of the Φ60X650 mm ingot along the axial direction. Observing the grain size and changing trend of the solidification structure of different degassing methods, the grain size at the bottom of the ingot was smaller than that at the top of the ingot and the middle of the ingot. However, it can be seen from the figure that the trend of grain size change was that the grain size was larger than 0.1 MPa under low-pressure conditions, and that the control group ingots (0.1 MPa ingots without any degassing treatment) in the solidified structure of a large number of pores were loose.

**Figure 5:**
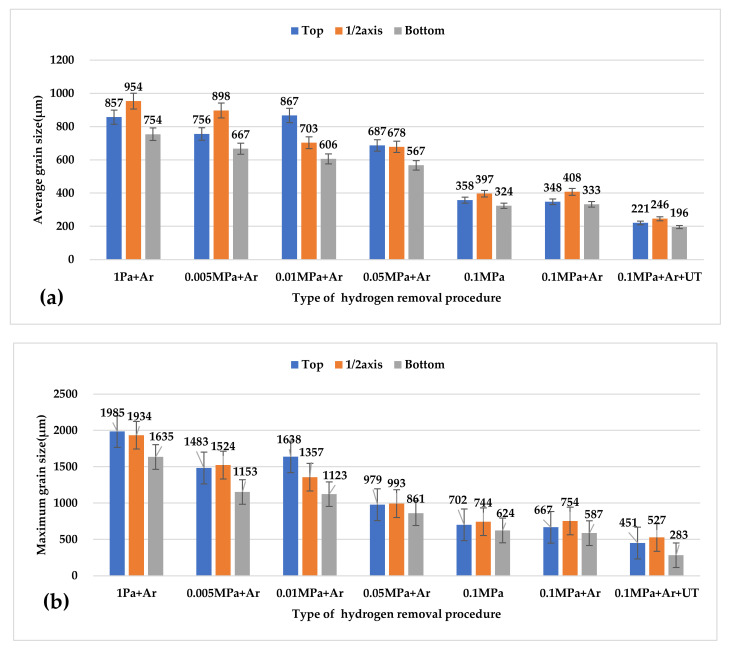
The average and maximum grain sizes of AA2195 alloys under different casting pressures: (**a**) the average grain size; (**b**) the maximum grain size.

**Figure 6:**
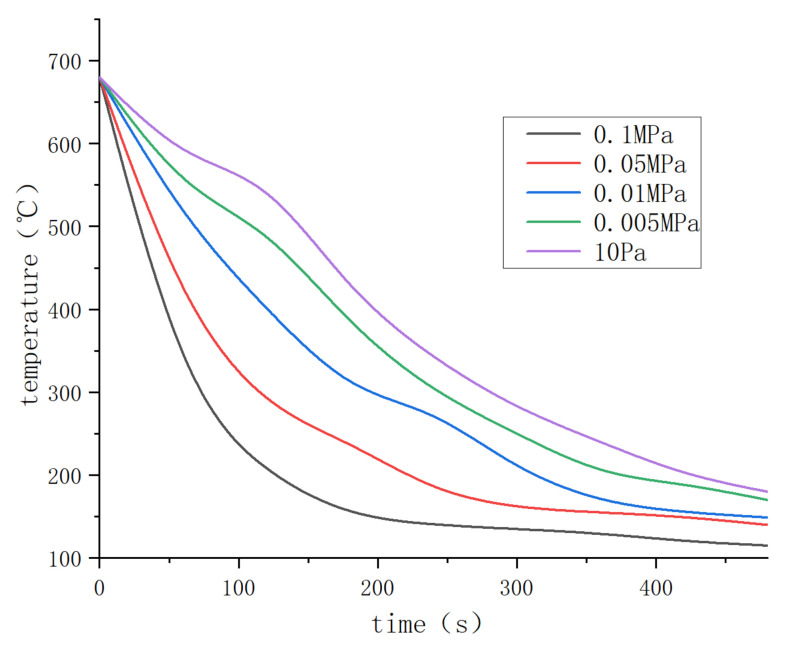
Cooling temperature curve of Φ60 mm 2195 Al–Li alloy ingot.

Figure 5 shows the statistics of the maximum grain size and average grain size in the metallographic structure of ingots at different positions. According to the statistical results in the figure, from the middle position of the ingot to the two ends of the ingot, the maximum grain size and the average grain size gradually decreased. Furthermore, as the argon pressure during casting increased, the size of the crystal grains also gradually decreased. The maximum particle size generally appeared in the middle position of the ingot. The average value in the middle position of the ingot with a pressure of 10 Pa was 953.70 μm, and the largest value was 1985.3 μm. With the gradual increase in argon pressure, the grains of the entire ingot size gradually reduced. Under the pressure of 0.1 MPa + Ar, the sum of the maximum particle size in the middle of the ingot was 754.10 μm. The average and maximum grain sizes of the control group were similar to those of the 0.1 MPa + Ar ingot. The average grain size gradually decreased from the center of 953 μm to 857.26 μm to the bottom of the ingot at 332.57 μm. The reason for this was that the pressure value in the vacuum chamber (10 Pa, 5000 Pa, 0.01 MPa, 0.05 MPa, and 0.1 MPa) was not changed until casting was completed. Under the condition of argon gas cooling with the same flow rate, the heat transfer medium in the furnace cavity was relatively thin under low pressure, and it could only be cooled by the furnace wall and the argon gas blown into the cooling chamber. Accordingly, the heat conduction efficiency was low, resulting in the ingots cooling relatively slowly at low pressure. To verify this, the temperature change curve of the ingot cooling was measured, as shown in Figure 6.

The cooling curve of the top of the ingot was obtained utilizing a thermocouple installed in the top of the mold cavity (Figure 1). It can be seen from Figure 6 that, as the pressure in the furnace cavity decreased, the rate of temperature drop continuously decreased. The cooling rate was faster within about 300 s. With the increase in air pressure, the cooling rate of the ingot under negative pressure was different in different periods of cooling. Upon a return to atmospheric pressure (0.1 MPa), the final cooling rate tended to be unchanged.

**Figure 7:**
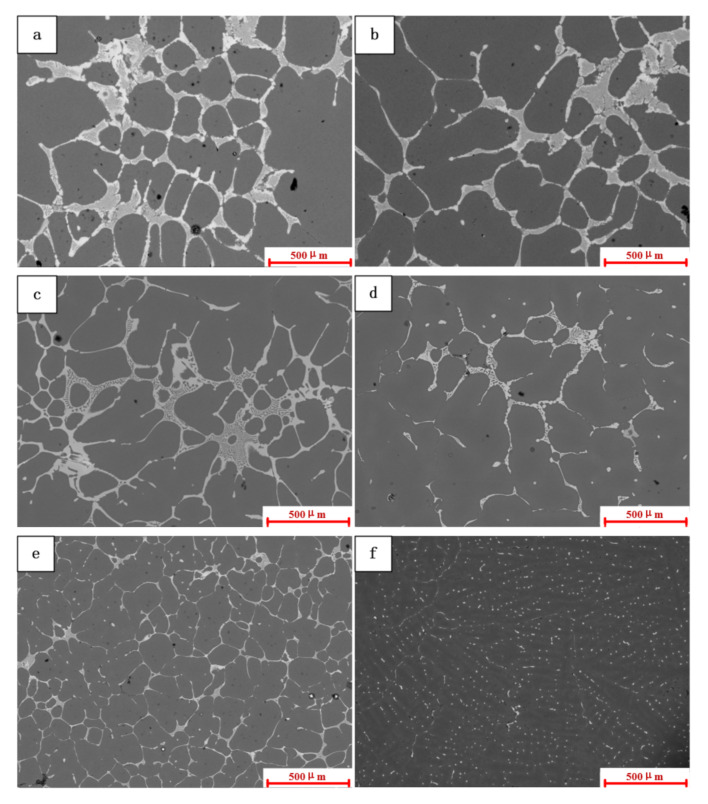
SEM images of the eutectic structure at the bottom of the ingot under different casting pressures: (**a**) 10 Pa + Ar; (**b**) 5000 Pa + Ar; (**c**) 10,000 Pa + Ar; (**d**) 50,000 Pa + Ar; (**e**) 0.1 MPa + Ar; (**f**) 0.1 MPa + Ar + UT.

Figure 7 shows the SEM images of the eutectic structure at the bottom of the 2195 Al-Li alloy ingot under different hydrogen removal procedures. It can be observed from Figure 7a–c that a large number of continuous eutectic structures gathered near the grain boundaries, presenting a coarse network structure. There was no discernible change trend in the morphology and distribution of eutectic structures in this area. The coarse eutectic structure in Figure 7d–f was reduced, especially in Figure 7f where the coarse eutectic structure on the grain boundary disappears, and the eutectic precipitation particles existed in the structure as circular discrete particles. When the material underwent tensile deformation, the coarsening of the eutectic structure was the initial location for cracks [15], causing a decrease in the mechanical properties of the ingot. This change was mainly due to the increased heat transfer efficiency of the ingot after the density of the cooling medium increased, whereby, in addition, a faster cooling rate inhibited the growth of the coarse eutectic structure in the 0.1 MPa + Ar + UT group, and the ultrasonic distribution of the components in the molten pool was more uniform and the generation of coarse eutectic structure was suppressed. Since the eutectic morphology of the control group was similar to that of the 0.1 MPa + Ar group, it is not discussed separately.

**Figure 8:**
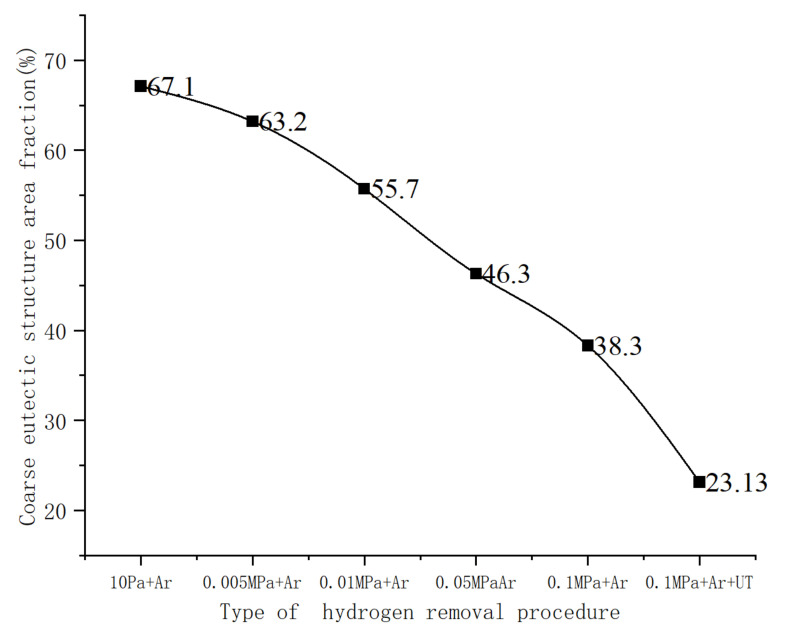
Area fraction of the coarsening eutectic phase.

Image-Pro Plus (IPP) software was used to quantitatively describe the distribution and position changes of the eutectic structure, as well as the area fraction of the coarse eutectic structure along the grain boundary region >20 μm^2^ and the area fraction of the eutectic structure at the bottom of the ingot under different casting pressures. At each sampling position, the statistical values of eight regions were averaged as the final result. It can be seen from Figure 8 that the area fraction of the eutectic structure on the grain boundary in the microstructure at the bottom of the ingot was in the range of 40–80%. Under the condition of 10 Pa, the area fraction of the eutectic phase was 67.1%, and, under the condition of 0.1 MPa, the area fraction was reduced to 42.4%. The changing trend of the area fraction of the eutectic structure in the ingot under different casting pressures is that, under the condition of higher pressure, the area fraction of the coarse eutectic was reduced; however, the experimental statistical results show that the distribution of the eutectic phase structure in the ingot was very uneven.

To characterize the size of the eutectic structure, the eutectic phase was measured using the software. Table 2 presents the average length and width of the eutectic phase structure of the ordinary 2195 Al–Li alloy ingot under different casting conditions. The average length of the crystalline structure was reduced from 77.5 μm under the condition of 10 Pa to 44.8 μm under the condition of 0.1 MPa + Ar, and the continuity of the eutectic structure was reduced by 46.1%. The average width was reduced from 25.2 μm under the condition of 10 Pa to 16.1 μm under the condition of 0.1 MPa + Ar, while the reduction in the eutectic structure at the width level reached 49.2%.

**Figure 9:**
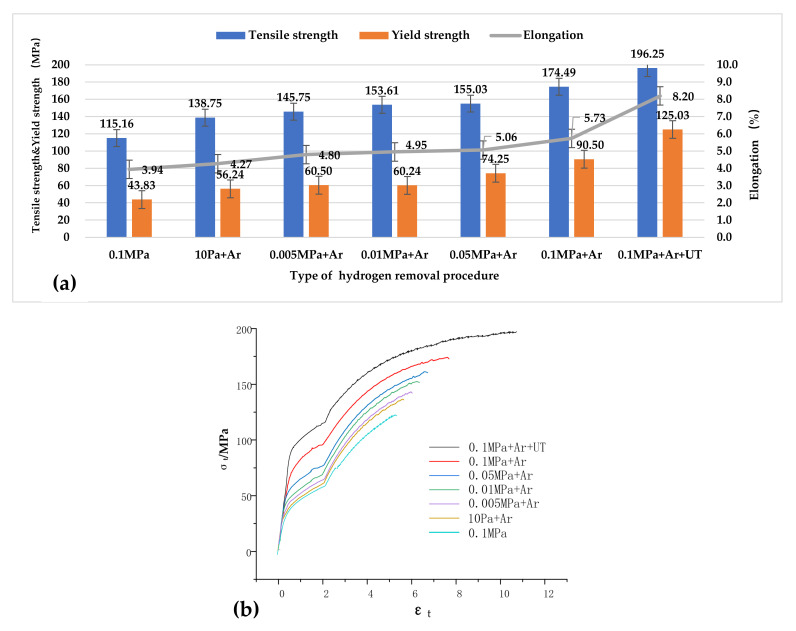
Mechanical properties of 2195 Al–Li alloys under different hydrogen removal procedures. (**a**) The mechanical properties of the value; (**b**) Stress-strain curve.

According to the test results in Figure 9, the mechanical properties of the ingots under different degassing conditions were obtained. The tensile strength of the ingot tensile sample was measured at 10 Pa. The average strength was 138.75 MPa, with a 4.27% elongation and a yield strength of 56.24 MPa, indicating brittle fracture characteristics. The average tensile strength of the sample was 196.25 MPa under 0.1 MPa pressure when also applying ultrasonic treatment, and the elongation was raised to 8.20%. Compared with the control group without any degassing treatment at 0.1 MPa, the mechanical properties of the degassed ingots were still improved. On the other hand, the yield strength of alloys treated with various degassing processes was very close; hence, it was not affected by degassing. In summary, degassing, particularly ultrasonic argon degassing, can significantly improve the mechanical properties of an as-cast alloy.

**Figure 10:**
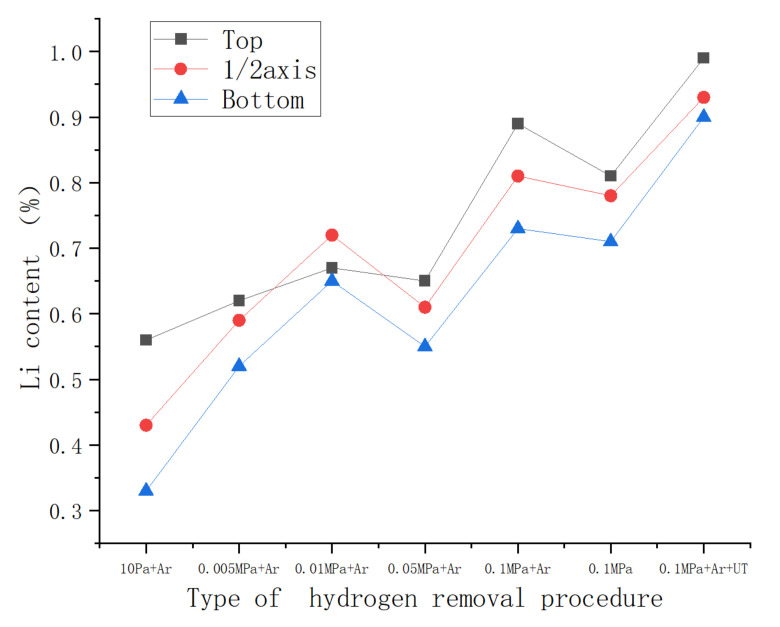
The content of the Li element at the axial sampling position of the Φ60 mm 2195 Al–Li alloy ingot.

Figure 10 shows the distribution of the Li element content in the three axial sampling areas of the ingot after the skin was removed. It can be seen from Figure 10 that in the axial direction of the ingot, the actual Li content of the ingot head was significantly higher than the average value of the ingot. Moreover, under different casting conditions, although the Li content was different due to burning loss and gasification, the general trend is that the head was higher than the middle and the bottom. Using an ingot with a casting pressure of 0.1 MPa as an example, the actual Li content at the bottom of the ingot was lower than the average value (0.817%), and the Li content was as low as 0.77%, which is lower than the standard value of 0.8% for the Li content specified by the alloy. The actual Li content and the average value of the middle part of the ingot were approximately 0.81%, whereas the actual Li content at the top of the ingot was significantly higher than the gap, at approximately 8%. From the perspective of the axial Li content distribution of the five groups of ingots, although the pressure conditions of the casting were different, the distribution of Li content in the axial direction of the ingot was the same. The basic law was that the content of the Li element was lower than the top of the ingot. The bottom of the ingot showed a stepped distribution. This shows that the distribution of Li elements in the ordinary 2195 Al–Li alloy ingot on a certain scale was very uneven, which had a certain degree of influence on the content and distribution of the eutectic structure in the ingot.

According to the data in Table 3, as the vacuum degree decreased, the hydrogen content in the ingot decreased, and the burning loss rate of the Li element increased accordingly. Under the condition of 10 Pa, the average burning loss rate of the ingot reached 56%. In the case of 0.1 MPa with ultrasound, the burning loss rate of the ingot was only one-tenth of that in the 10 Pa state, at 5.8%. The hydrogen content of the ingot was also less than 0.12 mL/100 g Al.

## 4. Discussion

### 4.1. Degassing of Al–Li Melt

It is well known that, for several metals, hydrogen can be effectively removed by purging the melt with finely dispersed argon gas bubbles. The principle is simply that hydrogen begins to accumulate in the insoluble gas as required for equilibrium with the dissolved concentration in the metal. It may be of considerable interest for aluminum to apply the simple model of hydrogen removal for other metal melts. The overall reaction between the bubble and the melt during purging should be divided into the following steps [16]: (1) transport of hydrogen from the melt to the melt/bubble phase border; (2) phase boundary reaction H = 1/2 H_2_; (3) gaseous hydrogen kept away from the melt/bubble phase transition.

Ultrasonic treatment is well known in metal melt processing for grain refinement, degassing, and purification [17,18]. Ultrasound’s postulated gas removal process mostly involves cavitation and rectification mass diffusion [19,20]. The ultrasonic wave causes alternating high pressure when it travels through the melt. Cavitation bubbles may emerge in the melt if the alternating pressure exceeds the cavitation threshold. There have been two suggested nucleating mechanisms for the cavities, while one assumes that there are already existing gas cavities in the liquid that act as nuclei, and the other assumes that the cavities develop spontaneously [21]. Non-wettable particles, particularly oxides in metal melts, might operate as a nucleation surface for cavities, according to the third theory. Because Al–Li melts include a substantial number of oxides, both of these methods are plausible.

At the same time, the reduction in hydrogen content of the ingot in the vacuum state is based on the solubility formula of hydrogen in the aluminum melt [22]:

(1) $$S=K\sqrt{P_{H_{2}}}$$

where *S* is the solubility of hydrogen in the melt, *K* is the dissolution constant, which is related to the temperature and the properties of the melt, and $P_{H_{2}}$ is th e partial pressure of hydrogen on the surface of the melt. Because $P_{H_{2}}$ under vacuum conditions is very low, the solubility of hydrogen is reduced, resulting in a significant drop in the content of hydrogen in the melt, while hydrogen overflows the melt as the pressure decreases.

The cavitation bubbles are expected to expand and collapse periodically under alternate pressure once they have nucleated. The dissolved hydrogen diffuses into the cavitation bubbles by “rectified mass diffusion” under positive pressure. Large bubbles can form quickly and float away from the metal surface, only to be replaced by new bubbles. In this way, hydrogen is continuously removed from the melt. Although ultrasonic degassing is effective, it can only handle a limited amount of molten metal.

**Figure 11:**
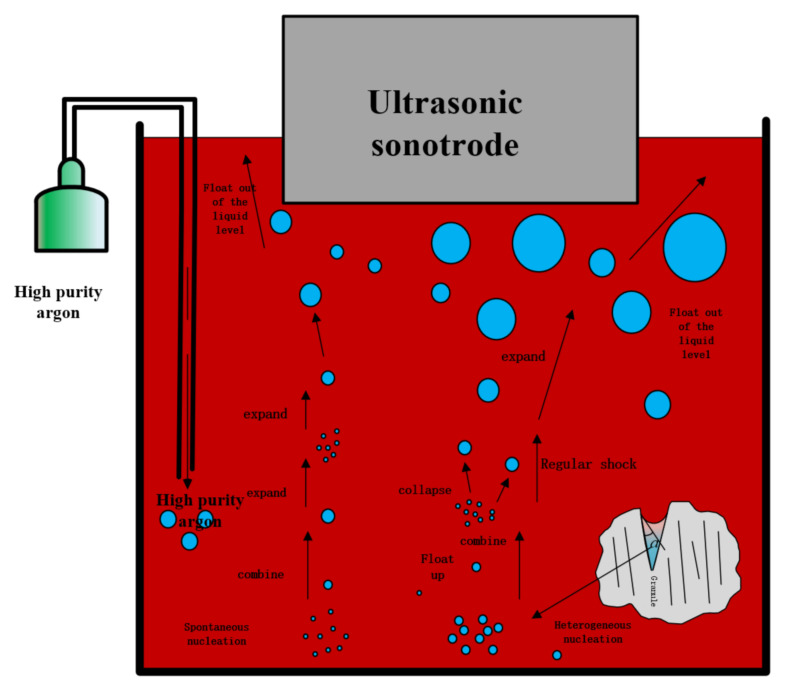
Diagram of the ultrasonic argon degassing process.

Bearing in mind those cases where high-intensity ultrasonic waves are injected simultaneously with purified argon, the major goal of the current study was to employ ultrasonic waves to dissolve and purify argon bubbles. The ultrasonic argon degassing process is depicted schematically in Figure 11. The argon gas is blasted out via the ultrasonic radiator’s core. It is thought that the ultrasonic field and the growing argon bubble should have a specific dynamic. Under the alternating pressure of ultrasonic vibrations, the purged argon bubbles can expand and compress again. These bubbles will not burst because argon is insoluble in the aluminum melt. They can be broken down into smaller pressures under high intensity and periodic pressure. The bubble surface area can then be increased because of this operation. Furthermore, ultrasonic-corrected mass diffusion may be applied to the melt/argon bubble interface, increasing hydrogen diffusion from the melt to the argon bubble. The sonic flow effect is also suggested to aid in the uniform distribution of refined argon gas bubbles throughout the melt [23,24].

High-intensity ultrasonic waves, on the other hand, produce a significant number of cavitation bubbles. The ultrasonic cavitation bubbles can ascend to the melt surface more easily with the help of argon bubbles. This implies that the ultrasonic degassing technique could also be used in the combing process. In other words—and as can be seen in Figure 2—the degassing efficiency was improved.

### 4.2. Degassing Process Refines Microstructures

Under various degassing settings, the state of the crystal grains and the T_2_ eutectic phase may be readily visible, as shown in Figure 7. Refining the structure grains by ultrasonic degassing is possible because cavitation enhances the nucleation mechanism [25]. Due to the slow cooling rate under vacuum conditions, the crystal grains of the ingot in the vacuum degassing treatment are larger than the ingot under the condition of 0.1 MPa. A refining agent was intentionally not introduced to the experiment. To better predict grain size, a solute suppression nucleation (SSN) model was recently published. When the solute diffusion curve (SDP) develops close to the previously established growing granules [26], the SDP of Al and Zn may form in untreated alloys due to poor convection and mass transfer. When the melt is purged with argon, the rising bubbles create significant mass transfer convection throughout the crucible. This procedure can homogenize the solute field, allowing the SSN effect to be ignored.

At the same time, when the “cold” argon bubbles rise from the melt’s bottom, they may cause supercooling, boosting the nucleation rate throughout the solidification process. Although the argon gas purged from the melt can effectively eliminate possible nucleation particles, the process takes considerable time to complete. As a result, the grain size is affected by the cooling rate during the solidification and nucleation processes.

In comparison to ultrasonic refinement, the grain refining impact of argon treatment is still restricted to 0.1 MPa. Ultrasound refines the voiceprint mostly owing to acoustic cavitation. A developed chain reaction of cavitation bubble nucleation, growth, and collapse is produced by alternating pressure [25]. The conversion of energy from the multiple collapsed holes into cumulative jets (up to 100 m/s), which introduce diverse physical and chemical processes into the melt [26], is critical. For example, better cavitation can speed up the wetting of particles in the melt, allowing them to function as an active heterogeneous nucleus. It can also simultaneously promote heat and mass transport. When ultrasonic and argon are both injected into the melt, the rising argon bubbles should affect ultrasonic cavitation, as evidenced by the microstructure difference illustrated in Figure 4.

### 4.3. Mechanical Properties Promoted by Degassing Process

As illustrated in Figure 9, ultrasonic degassing and grain refinement considerably enhanced the mechanical characteristics of the cast 2195 Al–Li alloy, particularly its ultimate strength and elongation. Mechanical performance may be improved for a variety of reasons, such as grain refinement, as seen in Figure 5. The yield strength of the 2195 Al–Li alloy is also known to be linearly proportional to the square root of the grain size [1,27], i.e., the Hall–Petch equation:

(2) $$\sigma_{y}=\sigma_{0}+kd^{-1/2},$$

where *σ_y_* is the yield strength of the material, *σ_0_* is the lattice friction of the material, *k* is the material constant, and *d* is the average grain size. From Figure 5, it can be seen that the average grain size of the sample was reduced from 363 μm to 221 μm after applying ultrasound. According to Equation (2), the yield strength was increased to 4.92 MPa. This is far removed from the test result data, indicating that the main change in the properties of the ingot was not caused by the crystal grains. Furthermore, the calculation findings demonstrate that, despite the varied grain sizes, the yield strength was fairly close for alloys that easily absorbed hydrogen. Because ultimate strength and grain size are exponentially related [28,29]., this degree of grain refinement will not result in a significant improvement in strength. As a result, grain refinement was not the primary cause of the increased final strength and elongation. The ultrasonic argon degassing alloy also offered the highest ultimate strength and the smallest grain size, also proving that grain refinement was not the main factor.

The ultimate strength and elongation of the as-cast 2195 Al–Li alloy are extremely susceptible to microporosity. A greater sensitivity leads to a coarser grain size [29,30]. As a result, the mechanical characteristics of the 2195 Al–Li alloy are highly dependent on microporosity. As mentioned in Section 4.1, the concentration of hydrogen has a direct relationship with microporosity. Micropore development is less likely to occur when the hydrogen level is low. As a result, as the alloy’s hydrogen level falls, the ultimate strength and elongation improve dramatically.

### 4.4. The Relationship between Li Element and Hydrogen Content

The addition of lithium to the Al–Li alloy increased the hydrogen absorption performance of the alloy approximately 50-fold. In traditional aluminum alloys, hydrogen accounts for more than 85% of the total gas dissolved in the aluminum melt. Therefore, under the same conditions, the proportion of hydrogen absorbed in the Al–Li alloy will be higher. When the hydrogen content in the melt reaches a certain level, very stable LiH impurities will be produced, which will cause a significant amount of burning of the Li element in the melt [31].

(3) $$H_{2}+2Li^{+}=2LiH$$

(4) $$2LiH+H_{2}O=Li_{2}O+2H_{2}$$

Moreover, the hydrogen pores and cracks that were generated increased stress corrosion sensitivity and led to hydrogen embrittlement; these defects seriously affected the mechanical properties of the alloy, as well as the development and use of ingots. Therefore, in the ingot preparation process, before adding Li and Mg elements, the aluminum melt in the molten pool should be refined to ensure that the hydrogen content in the melt is in a lower range before adding Li elements. Then, a second refinement is carried out before the casting is completed.

The reason for this is that, although Li and other alloying elements can be prevented from being oxidized under vacuum smelting conditions, these metal elements have a higher saturated vapor pressure; hence, evaporation loss will occur under vacuum smelting conditions, and the elements will evaporate. The rate can be described [32] according to the following expression:

(5) $$W=0.0263\sqrt{\frac{M}{T}}\left(-P+P_{i}\right),$$

where *W* is the evaporation rate, *P_i_* is the saturated vapor pressure of the element, *P* is the partial pressure of the element on the surface of the melt, *M* is the atomic weight of the element, and *T* is the temperature. Therefore, due to the high *P_i_* of the Li element, under low-pressure conditions, the evaporation rate *W* will increase greatly and the loss of Li element will increase sharply. Furthermore, lithium vapor has strong reducibility. Corrosion equipment causes hidden dangers both to the operation of the equipment and to safety.

According to the experimental data (Table 3), under the protection of argon gas, the application of ultrasound can simultaneously achieve the effect of increasing the content of Li in the ingot and reducing the content of hydrogen.

### 4.5. The Prospect of This Degassing Technology in Al–Li Melt

Ultrasonic argon degassing has been proven to be a fast-degassing method for large volumes of aluminum alloy melting. Furthermore, because the bubble size is substantially smaller than in a single argon degassing procedure, the melt surface turbulence is reduced. It should also be effective in a large number of Al–Li alloy melts. However, substantial systematic work is still required. Scholars have gradually recognized the importance of degassing for Al–Li alloys. This novel application of ultrasonic argon gas is very convenient for processing Al–Li alloy melts. In conclusion, the combination of ultrasonic and argon treatment for Al–Li alloy melts looks to have a promising future.

## 5. Conclusions

The 2195 Al–Li alloy melt was subjected to argon degassing, ultrasonic degassing, and various pressure vacuum degassing settings in this study. Al–Li alloys contain high hydrogen concentrations, which can deteriorate their mechanical properties. The degassing procedure considerably enhances the cast alloy’s mechanical characteristics, which is mostly due to dehydrogenation. A more efficient hydrogen elimination procedure is represented by ultrasonic argon treatment, which is capable of degassing and refining alloys. The ultrasonic breaking of argon bubbles is responsible for the excellent degassing efficiency. Ultrasound treatment may also result in the formation of a large number of cavitation bubbles in the melt. This can allow microstructure refinement. The reduction in hydrogen content also reduces the content and distribution of Li in the ingot.

## Figures and Tables

**Table 1:** Composition of AA2195 Al–Li alloy [12].

Alloy	Alloying Elements (wt.%)						
	Li	Cu	Mg	Ag	Zr	Mn	Al
2195	1.0	4.0	0.4	0.35	0.1	0.2	Bal.

**Table 2:** Average length and width of eutectic phase structure under different casting conditions.

	10 Pa + Ar	0.005 MPa + Ar	0.01 MPa + Ar	0.05 MPa + Ar	0.1 MPa + Ar	0.1 MPa + Ar + UT
Average length (μm)	77.5	75.3	70.1	55.4	44.8	27.21
Average width (μm)	25.2	23.4	22.6	19.2	16.1	5.28

**Table 3:** The average lithium burning loss rate and the average hydrogen content of 2195 Al–Li alloy ingots under various pressures.

	Li Content (wt.%)	Loss Rate (%)	Hydrogen Content (mL/100 g Al)
10 Pa + Ar	0.44	56.00%	0.111
0.005 MPa + Ar	0.577	42.30%	0.1207
0.01 MPa + Ar	0.667	33.30%	0.1523
0.05 MPa + Ar	0.603	39.70%	0.1773
0.1 MPa	0.776	22.40%	0.424
0.1 MPa + Ar	0.817	18.30%	0.2167
0.1 MPa + Ar + Ut	0.942	5.80%	0.118

## Data Availability

Data sharing is not applicable for this article.
